# Potential Serum HMGB1, HSP90, and S100A9 as Metastasis Predictive Biomarkers for Cancer Patients and Relevant Cytokines: A Pilot Study

**DOI:** 10.3390/ijms252413232

**Published:** 2024-12-10

**Authors:** Worawat Songjang, Chatchai Nensat, Wittawat Jitpewngarm, Arunya Jiraviriyakul

**Affiliations:** 1 Integrative Biomedical Research Unit (IBRU), Faculty of Allied Health Sciences, Naresuan University, Phitsanulok 65000, Thailand; worawats@nu.ac.th (W.S.); chatchaine@nu.ac.th (C.N.); 2 Department of Medical Technology, Faculty of Allied Health Sciences, Naresuan University, Phitsanulok 65000, Thailand; 3 Department of Cardio-Thoracic Technology, Faculty of Allied Health Sciences, Naresuan University, Phitsanulok 65000, Thailand; 4 Department of Medicine, Faculty of Medicine, Naresuan University, Phitsanulok 65000, Thailand; starplatinum07@hotmail.com

**Keywords:** metastasis predictive biomarker, DAMPs, HSP90, HMGB1, S100A9, cancer cytokines, cancer chemokines, TNM staging

## Abstract

Metastatic cancer is still one of the leading causes of death worldwide despite significant advancements in diagnosis and treatment. Biomarkers are one of the most promising diagnostic tools that are used alongside traditional diagnostic tools in cancer patients. DAMPs are intracellular molecules released in response to cellular stress, tissue injury, and cell death. There have been shown to be associated with worsening prognosis among such patients, and some DAMPs could potentially be used as predictive biomarkers of metastatic status. The goal of this study is to investigate DAMP expression and the probability that certain DAMPs could be predictive biomarkers of the metastatic stage in various cancer types. Forty cancer patients at Naresuan University Hospital, Thailand, were enrolled. Then, an investigation of HSP90, HMGB1, S100A9, and ATP expression and cytokine/chemokine profiling in serum was performed using an immunological-based assay. We assessed the predictive biomarker candidates and the association between DAMP expression and cytokines/chemokines using an ROC curve analysis and a correlation regression analysis. The results showed that HSP90 has strong potential as a metastatic predictive biomarker, with a cutoff value of 25.46 ng/mL (AUC 0.8207, sensitivity 82.61%, specificity 75.00%, 95% CI 0.6860–0.9553). This was followed by HMGB1 and S100A9, which exhibited sensitivity of 82.61 and 65.22%, and specificity of 68.75 and 56.25%, respectively. Interestingly, the candidate DAMPs negatively correlate with various serum cytokines, for example, HMGB1 vs. IL-15 (slope 88.05, R 0.3297, *p*-value 0.005), HMGB1 vs. IFN-γ (slope 2.235, R 0.3052, *p*-value 0.0013) and HSP90 vs. IFN-γ (slope 0.0614, R 0.2187, *p*-value 0.008), suggesting that they are highly elevated in advanced metastatic tumors, which is possibly associated with the immunomodulation effect. We postulated that HSP90, HMGB1, and S100A9 have the potential to be predictive biomarkers for supporting tumor metastasis categorization using histopathology.

## 1. Background

Cancer is one of the leading causes of death worldwide, with high incidence for decades. Even though current diagnosis and treatment have significantly improved over the past few decades, most morbidity and mortality are still a consequence of cancer metastasis, with the metastatic stage being considered the final stage of cancer [1]. There are various methods for diagnosing the cancer stage, such as imaging modalities and biopsy for histopathology; all diagnostic tools can helpfully determine the treatment plan for the patient [2]. However, the expeditious investigation of worsening from no distant metastasis to metastasis is still challenging.

Biomarkers are one of the promising diagnostic tools used as an early warning in a variety of diseases. Disease progression is a consequence of biomolecular fluctuations, some of which specifically reflect pathogenesis. In oncology, biomarkers have been used alongside traditional diagnostic methods due to rapid detection, less radiation/toxication exposure for the patient, no requirement of expensive clinical settings, and low cost burden [3,4,5]. However, developments in using biomarkers for distinguishing the severity of tumor progression in terms of metastasis in numerous cancer types are still limited.

Damage-associated molecular patterns (DAMPs) are the well-known danger signal that is released by stressed cells, injured cells or cell death, which can promote and aggravate the inflammatory response and cellular response, including tumorigenesis. The correlation between DAMPs and clinical symptoms of cancer has been reported by several studies as either a positive or a negative correlation, for example, high HMGB1 levels proportionally associated with poor overall survival in patients with advanced hepatocarcinoma treatment [6]. In breast cancer patients undergoing radiotherapy, the HSP70 significantly increased in the recurrent and metastatic group [7]. HSP90 expression is strongly associated with colorectal cancer, whose high expression reduces survival outcome [8]. Moreover, elevated levels of S100A8 and S100A9 expression correlate with diminished disease-free survival rates in cancer patients [9]. Interestingly, DAMPs have been also reported as having both good and bad consequences, such as tumor-promoting and antitumor biomolecules [10].

DAMPs have been elucidated as immunomodulators that can promote immune response through either direct stimulation or cytokine/chemokine-mediated activation, and some inflammatory cytokines have been linked to tumor progression [11]. Inflammatory cytokines have been mentioned as activators of both innate and adaptive immune cells that lead them to recognize and destroy cancer cells. However, some of them can play the role of tumor promotors, resulting in cancer cell proliferation and invasion [12]. Whether DAMPs induce tumor progression through cytokine-mediated immune cell activation or not, the study of the relationship between DAMPs and cancer-involved cytokines is still important.

This research aimed to investigate DAMP expression in the metastatic stage of various cancer types, including their validation as metastatic predictive markers and correlation between DAMPs and cancer-involved chemokine and cytokine profiling. HMGB1, HSP90, S100A9, and ATP were emphasized as the expression DAMPs in cancer patient serum. Our results suggested that HMGB1 and HSP90 could potentially serve as predictive biomarkers of metastatic status in cancer patients. Moreover, the DAMPs under investigation significantly correlate with several cytokines that could be used as prognostic markers of cancer immunotherapy treatment.

## 2. Results

### 2.1. Cancer Patients’ Characteristics

The basic clinical data are presented in Table 1. Forty patients participated in this study with the following characteristics: 55% male and 45% female, with an age ranging between 42 and 86 years; most of them had cholangiocarcinoma/gall bladder cancer (37.5%) and colorectal cancer (35%), whereas other types of cancer were recruited in this study, including breast cancer, small-cell lung carcinoma, hepatocellular carcinoma, bladder cancer, and nasopharynx and periampullary cancer (accounting for 2.5–7.5%). The average time from diagnosis to the collection of the blood sample was 6 months, with a range between 21 days and 25 months. Blood collection was performed in patients who underwent the blood sampling before starting the treatment and others who were undergoing treatment. Patients received chemotherapy according to first-line treatment, mostly cisplatin + gemcitabine, followed by cisplatin + gemcitabine + FOLFOX, cisplatin + 5-fluorouracil, carboplatin + paclitaxel, and paclitaxel + FOLFOX. Additional information of patients with tumor and chemotherapeutic treatment is shown in Table S3. Moreover, tumor staging was classified according to TNM Classification of Malignant Tumors, 8th edition. For M1 patients, most of them have CCA/gall bladder cancer and colorectal cancer. The metastasis site is mostly found in the liver, followed by nonregional lymph node, lung and peritoneum, and some presented with multiorgan metastasis; the frequency is nonregional lymph node with liver, bone, pericardium, soft tissue and lung, as summarized in Table 1.

### 2.2. Serum Candidate DAMPs in Cancer Patients with TNM Staging

**Figure 1:**
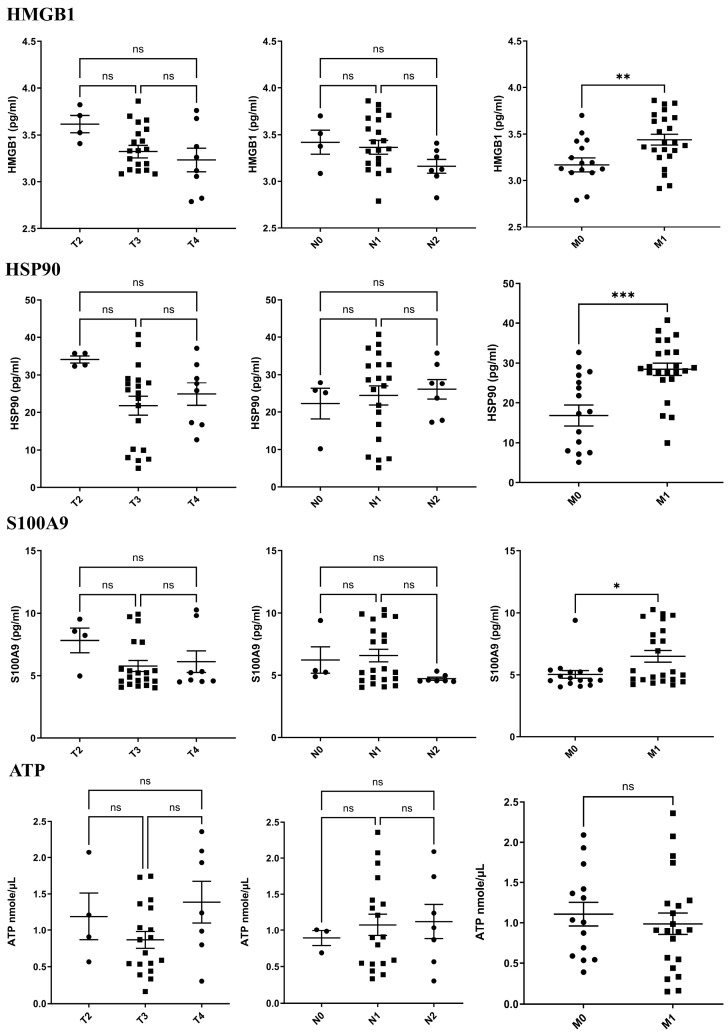
Serum candidate DAMP profiling according to TNM staging. Candidate DAMPs’ concentration levels in serum were determined with ELISA kit. All data are shown as individual mean values, M (mean values) ± SEM (standard error deviation). A significance threshold of *p*-value < 0.05 was considered as statistical significance. * *p*-value < 0.05; ** *p*-value < 0.01; *** *p*-value < 0.001; ns: *p* > 0.05.

To determine the levels of candidate DAMPs in cancer patients, serum HMGB1, HSP90, S100A9, and ATP were measured using ELISA and ATP assay kits. Data were partitioned according to the TNM staging of the cancer patients. The results are shown in Figure 1; the concentration of all candidate DAMPs is not significantly different in the T and N staging (Figure 1. Surprisingly, the spread of cancer cells in M1 patients exhibited highly significant levels of HMGB1, HSP90, and S100A9 when compared with M0 patients (Figure 1). The mean concentration of HMGB1 among M0 and M1 cancer patients was 3.16 ± 0.29 and 3.44 ± 0.28 ng/mL; for HSP90, it was 16.83 ± 10.48 and 28.46 ± 7.36 ng/mL; and for S100A9, it was 5.04 ± 1.26 and 6.49 ± 2.24 ng/m, respectively. However, there was no significant difference in serum ATP levels between M0 and M1 cancer patients.

### 2.3. Serum HMGB1, HSP90, and S100A9 as Biomarkers for Metastasis Differentiation

**Figure 2:**
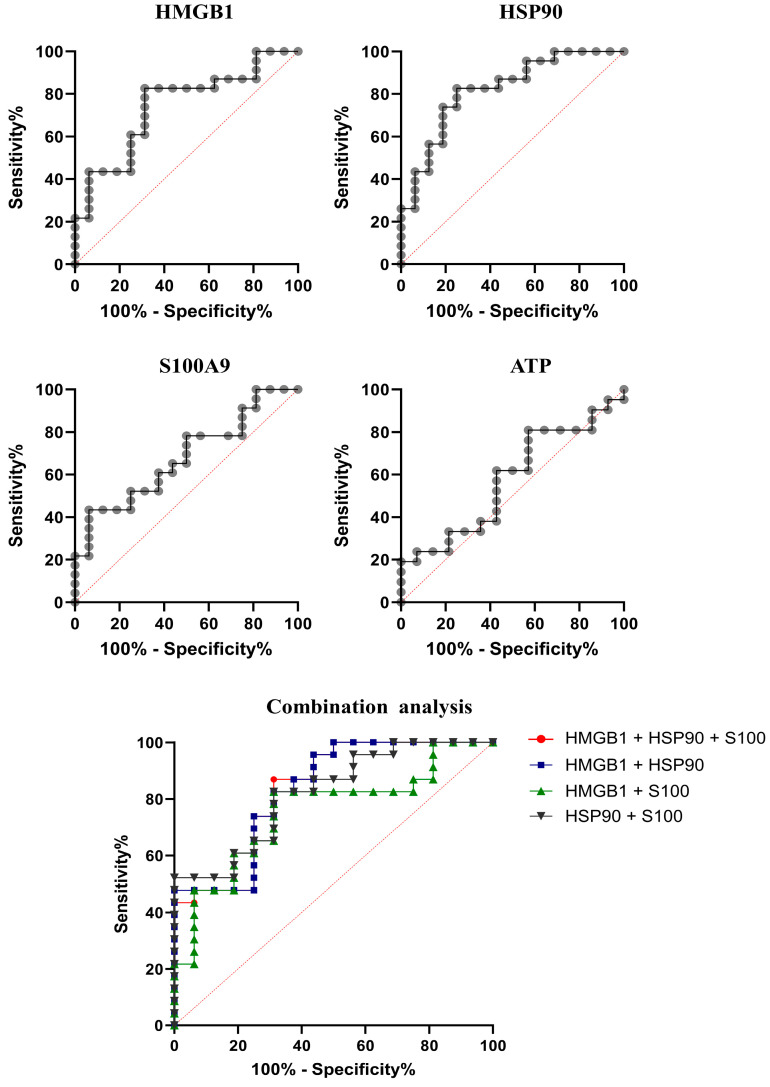
ROC analysis of candidate DAMPs for biomarkers of tumor metastatic status. ROC curve analysis of candidate DAMPs in predictive discrimination of M0 and M1 patients.

To evaluate the clinical assessment of DAMPs as candidate predictive biomarkers, the diagnostic efficiencies of serum HMGB1, HSP90, and S100A9 in M0 and M1 cancer patients were assessed with ROC curve analysis (Figure 2). A promising result was found for HSP90, which showed the most appropriate cutoff at 25.46 ng/mL with 82.61% sensitivity and 75.00% specificity. This was followed by HMGB1 and S100A9, which demonstrate sensitivity of 82.61 and 65.22% and specificity of 68.75 and 56.25%, respectively. Regarding area under the curve (AUC), HSP90 was the best at distinguishing the metastasis status of cancer patients, followed by HMGB1 and S100A9. When implementing these cut-offs with multi TNM considerations, the result showed that most patients with M1 staging have elevated serum HMGB1 and HSP90, higher than cut-offs, especially in serum HSP90, which seems to distinguish M1 with T2-4 and N1-2 from other M0 patients better than HMGB1 (Figure S1). Moreover, we used a combined analysis for DAMPs as candidate biomarkers for metastasis differentiation. However, the clinical sensitivity and specificity do not dramatically increase in the combination group. The analytical data are shown in Table 2.

### 2.4. Decrease in Serum Interferon Gamma and Interleukin-15 Associated with Metastasis Status and Relevant Correlation with Candidate DAMPs

**Figure 3:**
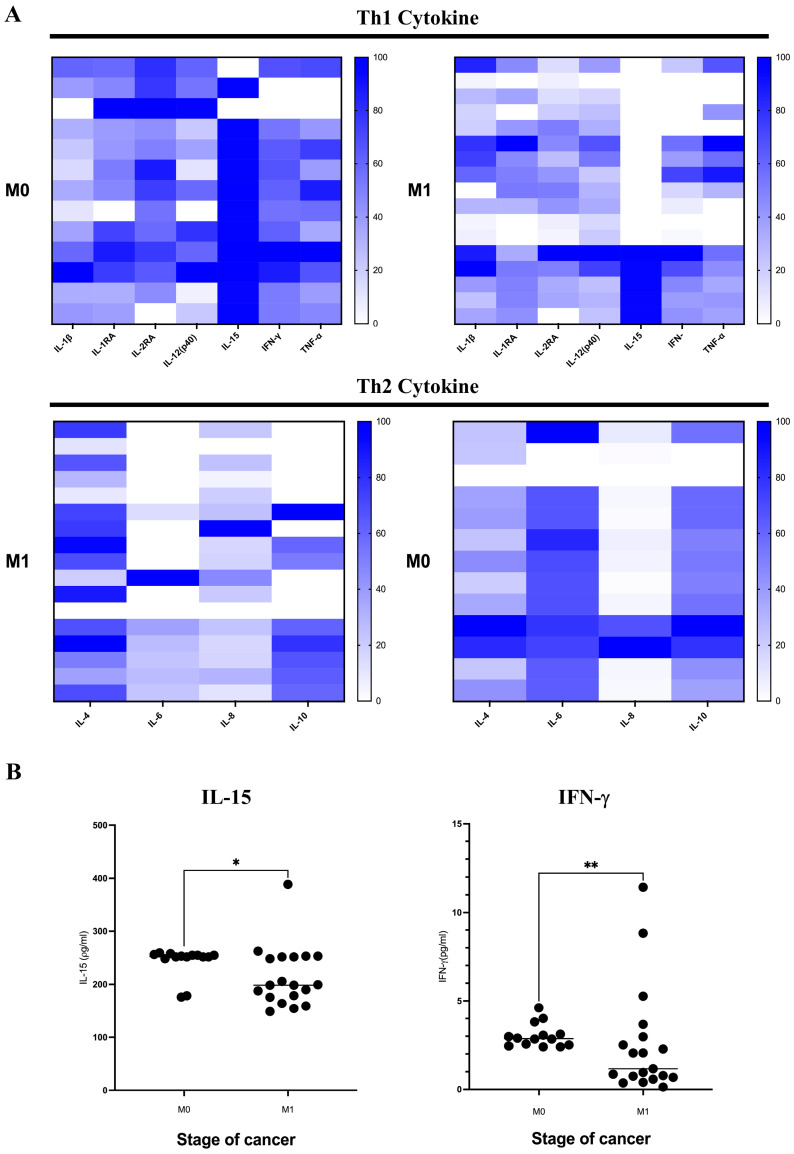
Cytokine and chemokine profiling of cancer patients’ serum according to tumor metastatic status. (**A**) Cytokines and chemokines were clustered according to their main deduced functions, including Th1/2 cytokines. (**B**) Analysis of selected cytokines, including IL-15 and IFN-γ in different stages of tumor metastasis. A significance threshold of *p*-value < 0.05 was considered as statistical significance. * *p*-value < 0.05; ** *p*-value < 0.01.

A panel of 48 cytokines was assessed by using Bio-plex Multiplex immunoassays (Bio-Rad, Hercules, CA, USA). The results indicated that only 38 cytokines/chemokines were defined at measurable levels in over 50% of the patients, whose cytokines/chemokines were included in the further investigation. The following ten cytokines were not discovered in over 50% of the patients: β-NGF, GM-CSF, GRO-a, IL-2, IL-3, IL-5, IL-7, IL-12(p70), IL-13, and MCP-3. The selection of cytokines was based on their significant role in regulating the tumor microenvironment, including promoting either the Th1 (IL-2RA, IL-12(p40), IL-15, TNF-α, IL-1β, IL-1RA, and IFN-γ) or the Th2 (IL-4, IL-6, IL-8, and IL-10) profile. Subsequently, the patients were categorized into two groups based on their M staging. The provided information is presented in Figure 1. This study revealed a notable decrease in the blood concentrations of IFN-γ and IL-15 among those classified as M1 patients in comparison to those classified as M0 patients, as shown in Figure 3. The present study aimed to examine the relationship between HMGB1, HSP90, S100, ATP, and cytokines in the serum of cancer patients. The findings indicated a negative correlation between HMGB1 and HSP90 and various cytokines, such as IFN-γ and IL-15, as indicated in Table 3.

## 3. Discussion

DAMPs are part of the protein response to cell injury, involved in the broad spectrum of cell death, from cell stress to autophagy, apoptosis, and necrosis [13,14]. DAMPs play many roles in molecular oncology, notably tumorigenesis, metastasis ability, and immunomodulation activity. The overgrowth of the tumor mass is accompanied by blood flow decrement and poor oxygen diffusion, resulting in hypoxic environment-causing cell injury and DAMP release [15]. DAMPs interact with specific receptors on the neighboring outer cell membrane, immune cells, and components of the extracellular matrix. These trigger a cascade of events that influence the tumor microenvironment. The HMGB1, HSP, and S100 families are considered well-known DAMPs because their function has been intensively studied over two decades. In particular, a high number of these DAMPs were found to be present during cell death when measured using either immune-based assay or proteomic analysis [16,17]. HMGB1, S100A9, and HSP90 were shown to be predictive and prognostic markers for distinguishing cancer patients from healthy controls in several cancer types such as mesothelioma, colorectal, gastric, and lung cancers [18,19,20,21]. In addition, these DAMPs were shown to be predictive and prognosis markers for cancer therapy [22,23]. Taken together, these candidate DAMP molecules exhibit the finest properties of cancer biomarkers.

In this study, we demonstrated that the major DAMPs HMGB1, HSP90, and S100A9 are promising predictive biomarkers for non-metastasis and metastasis discrimination. These DAMPs were released into the blood circulation of cancer patients and detected by drawing the blood and using the ELISA technique. The concentration ranges of HMGB, HSP90, and S100A9 were 2.48–3.87, 5.11–40.74, and 3.85–10.27 ng/mL, respectively. The concentration levels of these DAMPs were significantly elevated in M1 patients when compared with their M0 counterparts. The utilization of these DAMPs focused on their potential as predictive biomarkers according to the ROC curve analysis. Notably, three candidate DAMPs proved to be powerful predictors of metastatic tumors, with high sensitivity and specificity; HSP90 had the strongest predictive efficiency, as shown in Table 2. However, the combined ROC analysis of these DAMPs, such as HMGB1 with HSP90, HSP90 with S100A9, and combinations of all three, did not dramatically improve the predictive efficiency compared to single DAMPs alone, indicated by a slight increase in the area under the curve. It suggested that HMGB1 and HSP90 are promising predictive biomarkers for tumor metastasis discrimination, and that combined analysis of DAMP biomarkers is not necessary.

DAMP proteins are normally present in serum at normal concentration levels. In our study of HMGB1 as a predictive and prognostic biomarker of oncolytic immunotherapy, a concentration of 0.512 ng/mL was determined as the cutoff for categorizing patients into low and high HMGB1 baseline groups in various types of cancer [24]. A significant elevation of serum HMGB1 was found up to 24.7 ± 13.6 ng/mL in gastric cancer patients [25]. For HSP90, Fu Y et al. demonstrated that the basal level of serum HSP90α was 34.0 ± 20.8 ng/mL, and the concentration was dramatically increased in liver cancer patients with a mean concentration of 181.5 ± 105.4 ng/mL [26]. Interestingly, serum HSP90 exhibited potential as either a diagnostic or a stage predictive biomarker in liver cancer patients compared to alpha fetoprotein [26]. For S100A9, the baseline concentration was 10.05 (7.68–15.34) ng/mL in healthy controls and increased up to 22.32 (14.88–29.55) ng/mL in colorectal cancer patients [27]. Regarding different baseline concentration of DAMPs in our study, especially for HSP90 and S100A9, the first reason may be because of the capture antibody used in different ELISA kits. The second reason would be the differentiation of sample handling and storage. Although our results demonstrated the diverging baseline of the DAMPs, they precisely predicted the metastatic status of the cancer patients. In addition, this study decided to collect the data by limiting the time interval, obtaining various numbers of each tumor type that were non-homogenous with different etiologies. However, it has been reported that serum DAMPs were elevated and could be used as predictive markers in many types of cancer. Therefore, in this study, the random collection of serum in various tumors provides distinct serum DAMPs for use in real clinical settings.

Although unrelated to metastatic prediction, we investigated the role of candidate DAMPs in various conditions including lymph node invasion status, tumor size, and response for chemotherapy prediction (Supplementary Materials). However, the predictive efficacy of serum DAMPs was not strong enough to distinguish these conditions. The necrotic core of an excessive growth tumor and dying cells from chemotherapy are the main causes of DAMP release [15]. Moreover, DAMPs are necessary to promote cancer cell metastasis through molecular mechanisms, such as cell migration, invasion, adhesion, and EMT processes [10]. Hence, these may explain how DAMPs are elevated in metastasis cancer patients. The ideal properties of biomarkers should be present in accessible samples such as blood and urine with measurable concentration, specifically those released from the tumor site. Moreover, metastasis primitive biomarkers should be present with high sensitivity for true recruitment of metastatic patients and effective treatment planning. These match the candidate DAMPs in our demonstration that were specifically released from cancer cells and measured in the blood circulation, and were notably reflected in different stages of the tumor metastasis.

The transition of tumors from an early stage to a late stage is characterized by a multitude of cellular, biochemical, and molecular processes, which encompass the dysregulation of several growth factors. In the current investigation, the levels of IFN-γ and IL-15 were significantly decreased throughout the M1 stage, suggesting the potential clinical significance of this particular marker. It is well known that these cytokines critically control tumor progression via induction of immune surveillance. IFN-γ-induced cancer cell death mediated by either cell senescence or apoptosis led to high sensitivity of cancer treatment, inhibited regulatory T cells, and induced M1 macrophage polarization, resulting in strong antitumor immune responses [28]. Notably, IFN-γ inhibits tumor metastasis mediated by overexpression of fibronectin-1 [29]. As well as IFN-γ, IL-15 inhibits tumors by regulation of CD8^+^ T cells and NK cells, but research is lacking on IL-15′s association with the induction of breast cancer metastasis [30,31]. Taken together, this explains how the low concentration of IFN-γ and IL-15 in M1 patients is correlated with tumor metastasis. In addition, we conducted an examination of cytokine and DAMP expression by categorizing patients and using correlation regression analysis. A negative correlation was seen between HMGB1 and HSP90 with IFN-γ and IL-15, suggesting that the production of cytokines is associated with DAMP concentration levels.

There are some limitations of this study; the aforementioned findings were obtained from a small sample size, hence characterizing this as a *pilot study*. This might be improved by incorporating an additional cohort to provide a full range of predictive biomarkers for patients diagnosed with cancer. Moreover, we recruited all types of cancer, which may cause non-homogeneous data, especially for the serum DAMPs’ baseline concentration. As mentioned above, different studies in distinct tumor types show a non-similar baseline of DAMP concentration. Although we demonstrated a small sample size with various types of cancer, the overall data could reveal promising predictive efficiencies for metastatic discrimination in cancer patients. Moreover, we focused on candidate DAMPs including HMGB1, HSP90, and S100A9; other DAMPs should be considered for study of predictive effectiveness, such as mitochondrial DAMPs, nuclear, and other extracellular DAMPs. This could be achieved with proteomic profiling of DAMPs for large-scale data analysis [16,17]. Moreover, the effectiveness of candidate DAMPs should be further compared with current metastasis predictive biomarkers for demonstration in clinical utilization.

In conclusion, we demonstrated the predictive effectiveness of candidate DAMP biomarkers for discrimination between M0 and M1 cancer patients. We focused on HMGB1, HSP90, and S100A9, of which HSP90 was the best metastatic predictive marker, followed by HMGB1 and S100A9. Moreover, the candidate DAMPs showed a trend of negative correlation with serum cytokine, for example, IL-15 and IFN-γ, which strongly decreased in metastasis patients. This preliminary study provided essential evidence of DAMP predictive biomarkers for supporting tumor metastasis categorization using histopathology.

## 4. Methods

### 4.1. Cancer Patient Enrollment and Sample Collection

In this study, we recruited patients with all types of cancer, who received follow-up and underwent chemotherapy treatment at Naresuan University Hospital between February and August 2022. Cancer was diagnosed based on imaging modalities or serum biomarkers, and further confirmed with histopathology. All patients gave written informed consent, and this study was approved by the Human Research Ethics Committee according to the Declaration of Helsinki, the *Belmont Report*, CIOMS guidelines, and ICH-GCP (IRB No. 611/62). The information received from the case record form includes sex, age, type of cancer, date of diagnosis and treatment history of chemotherapy. Peripheral blood samples were collected as clotted blood before receiving chemotherapy. Serum was collected using centrifugation at 1000× *g* for 10 min and stored at −80 °C.

### 4.2. HMGB1, HSP90, and S100A9 Determination

HMGB1, HSP90, and S100A9 measurement was carried out using an ELISA kit ElabScience^®^ (Houston, TX, USA); the catalog numbers are E-EL-H1554, E-EL-H1864, and E-EL-H6197, respectively. Frozen samples were thawed at room temperature, and the concentration of candidate DAMPs was analyzed according to the manufacturer’s recommendations. Then, 100 μL of sample standard was pipetted into a coated ELISA plate and incubated for 90 min at 37 °C. Samples were removed, washed and subsequently supplemented with detection antibody before incubation for 1 h. After that, HRP conjugate solution was added and incubated for another 30 min. The reaction was stimulated with substrate for 30 min and stopped using stop solution, and the optical density was measured with an ELISA plate reader at 450 nm. The values of candidate DAMPs were analyzed by plotting the standard curve for the obtained linear equations.

### 4.3. Adenosine Triphosphate (ATP) Measurement

ATP measurement was performed in this study using an ATP Colorimetric Assay Kit (Sigma-Aldrich, St. Louis, MO, USA). Following the manufacturer’s protocol, 50 μL of patients’ serum was mixed with working reagent at a 1:1 ratio and incubated at room temperature for 30 min in the dark. Optical density was measured at 570 nm. The value was analyzed with standard curve and linear equations of standard ATP.

### 4.4. Cytokine Profiling of Cancer Patients

Before being analyzed, serum samples were obtained and kept at −80 °C. The aliquots used in the multiplex chemokine test had not undergone prior thawing. We quantified the levels of various cytokines and growth factors including FGF, Eotaxin, G-CSF, GM-CSF, IFN-γ, IL-1β, IL-1ra, IL-1α, IL-2α, IL-3, IL-12(p40), IL-16, IL-2, IL-4, IL-5, IL-6, IL-7, IL-8, IL-9, GRO-α, HGF, IFN-α2, LIF, MCP-3, IL-10, IL-12(p70), IL-13, IL-15, IL-17A, IP-10, MCP-1(MCAF), MIG, β-NGF, SCF, SCGF-β, SDF-1α, MIP-1α, MIP-1β, PDGF-BB, RANTES, TNF-α, VEGF, CTACK, MIF, TRAL, IL-18, M-CSF, and TNF-β with the Bio-plex Pro^TM^ Human Cytokine Screening 48-Plex Panel using the Bio-Plex200 (Bio-Rad Laboratories, Hercules, CA, USA) as per the manufacturer’s instructions. The complete names of cytokines are provided in Supplementary Table S2.

### 4.5. Data Analysis and Statistics

Statistical analysis was performed utilizing GraphPad Prism 10.0 software, with the data presented as mean values accompanied by standard errors (SEs). Receiver operating characteristic (ROC) curves were formulated to evaluate sensitivity, specificity and their corresponding areas under the curve (AUCs) while considering a 95% confidence interval (CI). Optimal diagnostic cutoff values were determined by maximizing the combined measurements of sensitivity and specificity. To assess the diagnostic performance of combined DAMP markers, the models were analyzed with logistic regression. After fitting, the new variable predicted probability (P) was subjected to ROC analysis. Variations in assessing the efficacy among cancer patients were analyzed using parametric and non-parametric tests regarding the distribution of data. A significance threshold of *p*-value < 0.05 (two-tailed) was considered as statistical significance.

## Figures and Tables

**Table 1:** Characteristics of cancer patients included in this study [median (min–max) or n (%)].

Patient Characteristics	Cases (*n* = 40)
Sex	
Male	22 (55%)
Female	18 (45%)
Age, years	63.5 (42–86 years)
NA	2 (5%)
<50	2 (5%)
50–59	5 (12.5%)
60–69	21 (52.5%)
70–79	7 (17.5%)
≥80	3 (7.5%)
Type of cancer	
Bladder	1 (2.5%)
Breast	3 (7.5%)
CCA/gall bladder	15 (37.5%)
Colorectal	14 (35%)
Hepatocellular carcinoma	2 (5%)
Small-cell lung carcinoma	3 (7.5%)
Nasopharynx	1 (2.5%)
Periampullary	1 (2.5%)
T staging	
NA	1 (2.5%)
Tx	7 (17.5%)
T0	0 (0%)
T1	0 (0%)
T2	4 (10%)
T3	20 (50%)
T4	8 (20%)
N staging	
NA	1 (2.5%)
Nx	7 (17.5%)
N0	4 (10%)
N1	21 (52.5%)
N2	7 (17.5%)
N3	0 (0%)
M Staging	
NA	1 (2.5%)
M0	16 (40%)
M1	23 (57.5%)
Site of metastasis	
Liver	7 (30%)
Nonregional lymph node	5 (22%)
Lung	4 (17%)
Peritoneal metastasis	1 (4%)
Multiorgan metastasis (Liver, Nonregional lymph node, bone, Pericardium, Soft tissue, Lung)	6 (26%)

CCA: cholangiocarcinoma; NA: not applicable; Nx: main tumor cannot be measured.

**Table 2:** Cutoff and essential information for ROC analysis.

Biomarkers	Area Under Curve (AUC)	95% CI	Cutoff (ng/mL)	Sensitivity(%)	Specificity(%)
HMGB1	0.7418	0.5831–0.9006	3.245	82.61	68.75
HSP90	0.8207	0.6860–0.9553	25.46	82.61	75.00
S100A9	0.6793	0.5114–0.8473	4.779	65.22	56.25
HMGB1 + HSP90	0.8315	0.7013–0.9617	NA	73.91	75.00
HMGB1 + S100A9	0.7554	0.6008–0.9101		78.26	68.75
HSP90 + S100A9	0.8027	0.6914–0.9499		73.91	68.75
HMGB1 + HSP90 + S100A9	0.8315	0.7004–0.9626		73.91	75.00

**Table 3:** Correlation regression analysis of DAMPs and cytokines from cancer patients’ serum.

	Slope	R Squared	*p*-Value
HMGB1			
Beta-NGF	−4.285	0.6078	<0.0001
IFN-γ	−2.235	0.3052	0.0013
IL-2	−3.074	0.3351	0.0005
IL-6	−0.8601	0.1393	0.0461
IL-7	−5.661	0.1733	0.0199
IL-10	−6.649	0.2926	0.0012
IL-12(p70)	−1.681	0.3906	0.0001
IL-15	−88.05	0.3297	0.0005
RANTES	−324.2	0.1490	0.0320
SCF	48.87	0.1694	0.0193
VEGF	−155.8	0.4178	<0.0001
HSP90			
Beta-NGF	−0.0854	0.2254	0.0060
IFN-γ	−0.0614	0.2187	0.0080
IL-2	−0.0699	0.1650	0.0211
IL-12(p70)	−0.0306	0.1283	0.0407
VEGF	−3.159	0.1707	0.0169
S100			
LIF	13.43	0.1384	0.0360
SCF	−16.40	0.1444	0.0319
ATP			
HGF	−92.19	0.1461	0.0338
IL-2Ra	−17.93	0.1746	0.0173
IL-8	−6.033	0.1776	0.0228
IL-16	−9.499	0.1814	0.0151
IP-10	−24.35	0.1672	0.0224
MIF	−61.32	0.1940	0.0132

## Data Availability

The original data in this study are included in the article/Supplementary Materials. Further inquiries can be directed to the corresponding authors.

## Supplementary Materials

The following supporting information can be downloaded at: https://www.mdpi.com/article/10.3390/ijms252413232/s1.
