# Supplementary material for: Potential Serum HMGB1, HSP90, and S100A9 as Metastasis Predictive Biomarkers for Cancer Patients and Relevant Cytokines: A Pilot Study
Source: Int J Mol Sci. 2024 Dec 10;25(24):13232. doi: 10.3390/ijms252413232 (PMC11675498; doi:10.3390/ijms252413232)
Supplement: Supplementary file 1 [file ijms-25-13232-s001.zip › ijms-3275748-supplementary.pdf]

Table S1. Concentration of candidates DAMPs.

Patients ID	Cancer type	staging			HMGB1 (pg/mL)	HSP90 (pg/mL)	S100A9 (pg/mL)
		T staging	N staging	M staging			
A001	CCA/gall bladder	T2	N1	M1a	3.822	32.661	8.219
A002	CCA/gall bladder	T2	N1	M1a	3.707	32.352	9.513
A003	CCA/gall bladder	T3	N1	M1a	3.333	40.743	7.680
A004	CCA/gall bladder	T3	N1	M1a	3.561	28.625	7.716
A005	CCA/gall bladder	T4	N1	M1a	3.761	29.065	9.809
A006	CCA/gall bladder	T4	N1	M1a	3.676	37.077	10.273
A007	CCA/gall bladder	T2	N1	M1a	3.526	35.748	8.546
A008	CCA/gall bladder	T3	N0	M0	3.700	25.124	9.387
A009	CCA/gall bladder	T3	N1	M1a	3.659	38.076	9.718
A010	HCC	TX	NX	M1a	3.832	29.355	6.927
A011	CCA/gall bladder	T3	N1	M1a	3.862	25.978	9.929
A012	CCA/gall bladder	T4	N0	M1	3.378	25.798	5.247
A013	CCA/gall bladder	T3	N1	M0	3.348	29.035	4.737
A015	HCC	T3	N0	M0	3.513	27.846	4.890
A016	Colorectal	T4	N2	M0	2.825	17.277	4.543
A017	Bladder cancer	Tx	N1	M1a	3.324	19.964	4.822
A018	Breast	T4	N2	M1a	3.260	27.676	5.331
A019	Colorectal	T3	N1	M0	3.123	26.967	5.399
A020	Colorectal	T3	N1	M0	3.083	7.157	5.519
A021	Colorectal	T3	N2	M0	3.116	17.806	4.562
A022	Nasopharynx	T4	N1	M0	2.789	12.741	4.578
A023	Colorectal	T4	N1	M1b	3.118	16.707	4.656
A024	Colorectal	T3	N1	M0	3.436	7.546	5.221
A025	Colorectal	T3	N1	M0	2.477	5.109	5.234
A026	Periampullary CA	T3	N0	M0	3.084	10.204	5.380
A027	Lung	T2	N2	M1b	3.410	35.728	4.977
A028	Breast	Tx	Nx	M1b	3.246	27.786	4.633
A029	Colorectal	T3	N1	M0	3.425	3.802	4.043
A030	Colorectal	T3	N1	M0	3.192	32.691	4.085
A031	Colorectal	T3	N2	M1a	3.329	27.586	4.617
A032	Small cell	Tx	Nx	M1a	3.361	27.646	4.990
A033	Breast	T3	N1	M0	3.187	21.792	4.169
A034	Colorectal	T3	N0	M1a	3.478	23.547	5.896
A035	CCA/gall bladder	Tx	Nx	M1b	3.478	25.635	5.748
A036	CCA/gall bladder	Tx	Nx	M1b	3.147	19.854	5.127
A037	Colorectal	Tx	Nx	M1b	3.589	16.875	4.789
A038	Lung	T4	N2	M1b	3.369	18.596	5.897
A039	Colorectal	T3	Nx	M1b	3.784	19.746	5.412
A040	Colorectal	T3	N1	M0	2.368	15.968	3.876
A041	Colorectal	T3	N2	M0	2.898	10.745	4.123

Table S2. Full names of analyte in the Bio-plex Pro™ Human Cytokine Screening 48-Plex Panel

Analyte	Gene name (HGNC)	Protein name (Uniprot)
FGF	FGF	Fibroblast growth factor
Eotaxin	CCL11	Eotaxin, C-C motif chemokine 11
G-CSF	CSF3	Granulocyte colony-stimulating factor
GM-CSF	CSF2	Granulocyte-macrophage colony-stimulating factor, Colony stimulating factor 3
IFN-γ	IFNG	Interferon gamma
IL-1β	IL1B	Interleukin-1 beta
IL-1ra	IL1ra	Interleukin-1 receptor antagonist
IL-1α	IL1A	Interleukin-1 alpha
IL-2α	IL2A	Interleukin-2 alpha
IL-3	IL3	Interleukin-3
IL-12(p40)	IL12B	Interleukin-12 subunit beta
IL-16	IL16	Interleukin-16
IL-2	IL2	Interleukin-2
IL-4	IL4	Interleukin-4
IL-5	IL5	Interleukin-5
IL-6	IL6	Interleukin-6
IL-7	IL7	Interleukin-7
IL-8	CXCL8	Interleukin-8
IL-9	IL9	Interleukin-9
GRO-α	CXCL1	Growth-regulated alpha protein, C-X-C motif chemokine ligand 1
HGF	HGF	Hepatocyte growth factor
IFN-α2	IFNA2	Interferon alpha 2
LIF	LIF	Leukemia inhibitory factor
MCP-3	CCL7	C-C motif chemokine 7, Monocyte chemoattractant protein 3
IL-10	IL10	Interleukin-10
IL-12(p70)	IL-12(p70)	Interleukin 12p70
IL-13	IL13	Interleukin-13
IL-15	IL15	Interleukin-15
IL-17A	IL17A	Interleukin-17A
IP-10	CXCL10	C-X-C motif chemokine 10, Interferon gamma-induced protein 10
MCP-1(MCAF)	CCL2	C-C motif chemokine 2, Monocyte chemoattractant protein 1
MIG	CXCL9	C-X-C motif chemokine 9, Monokine induced by gamma interferon
β-NGF	NGF	Beta-nerve growth factor
SCF	KITLG	Kit ligand, Stem cell factor
SCGF-β	SCGF-β	Serum stem cell growth factor beta
SDF-1α	CXCL12	Stromal cell-derived factor 1 alpha
MIP-1α	CCL3	C-C motif chemokine 3, Macrophage inflammatory protein 1-alpha
MIP-1β	CCL4	C-C motif chemokine 4, Macrophage inflammatory protein 1-beta
PDGF-BB	PDGF-BB	Platelet derived growth factor BB

RANTES	CCL5	Regulated upon activation, normal T cell expressed and presumably secreted
TNF-α	TNF	Tumor necrosis factor
VEGF	VEGF	Vascular endothelial growth factor
CTACK	CCL27	Cutaneous T cell-attracting chemokine
MIF	MIF	Macrophage migration inhibitory factor
TRAL	TNFSF10	TNF ligand superfamily member 10, TNF-related apoptosis-inducing ligand
IL-18	IL18	Interleukin-18
M-CSF	CSF1	Macrophage colony-stimulating factor 1
TNF-β	LTA	Lymphotoxin-alpha

Table S3 Additional information of patients with tumor and chemotherapeutic treatment.

Patients ID	M stage	Cancer type	Comorbidity	Chemotherapy (Yes/NO)	Adjuvant/ Palliative	Name of Drugs
A001	M1a	CCA/gall bladder	-	Yes	Palliative	Gem+Cis
A002	M1a	CCA/gall bladder	HT, DLP	Yes	Palliative	Gem+Cis
A003	M1a	CCA/gall bladder	HT, DM type 2, Pulmonary embolism	Yes	Palliative	Gem+Cis
A004	M1a	CCA/gall bladder	-	Yes	Palliative	Gem+Cis
A005	M1a	CCA/gall bladder	HT, DLP, CKD, Melanoma	Yes	Palliative	Gem+Cis
A006	M1a	CCA/gall bladder	Thalassemia	Yes	Palliative	Gem+Cis
A007	M1a	CCA/gall bladder	-	Yes	Palliative	Gem+Cis
A008	M0	CCA/gall bladder	-	Yes	Palliative	Gem+Cis+FOLFOX
A009	M1a	CCA/gall bladder	-	Yes	Adjuvant	Gem+Cis+FOLFOX
A010	M1a	HCC	Cirrhosis	Yes	Palliative	FOLFOX
A011	M1a	CCA/gall bladder	HT	NO	Palliative	Gem+Cis
A012	M1	CCA/gall bladder	-	Yes	Adjuvant	Gem+5FU
A013	M0	CCA/gall bladder	HT	Yes	Palliative	Gem+Cis
A015	M0	HCC		Yes	Palliative	Atelozumab Bevacizumab
A016	M0	Colorectal		Yes	Adjuvant	-
A017	M1a	Blabber cancer		Yes	Palliative	NA
A018	M1a	Breast		Yes	Palliative	Paclitaxel
A019	M0	Colorectal		Yes	Adjuvant	-
A020	M0	Colorectal		Yes	Adjuvant	-
A021	M0	Colorectal		Yes	Adjuvant	-

A022	M0	Nasopharynx		Yes	Adjuvant	-
A023	M1b	Colorectal		Yes	Palliative	NA
A024	M0	Colorectal		Yes	Adjuvant	-
A025	M0	Colorectal		Yes	Adjuvant	-
A026	M0	Periampullary CA	HT	Yes	Adjuvant	NA
A027	M1b	Lung	-	Yes	Palliative	Carbo+ Paclitaxel
A028	M1b	Breast	-	NO	-	-
A029	M0	Colorectal	HT, DLP	NO	-	-
A030	M0	Colorectal	DM type 2	NO	-	-
A031	M1a	Colorectal	-	Yes	Palliative	NA
A032	M1a	Small cell	-	NO	-	-
A033	M0	Breast	-	NO	-	-
A034	M1a	Colorectal	HT	NO	-	-
A035	M1b	CCA/gall bladder	HT, DM type 2	Yes	Palliative	NA
A036	M1b	CCA/gall bladder	HT	Yes	Palliative	Gem+Ciapatin
A037	M1b	Colorectal	-	Yes	Palliative	FOLFOX
A038	M1b	Lung	HT	Yes	Palliative	Carboplatin
A039	M1b	Colorectal	CKD	NO	-	-
A040	M0	Colorectal	-	NO	-	-
A041	M0	Colorectal	-	NO	-	-

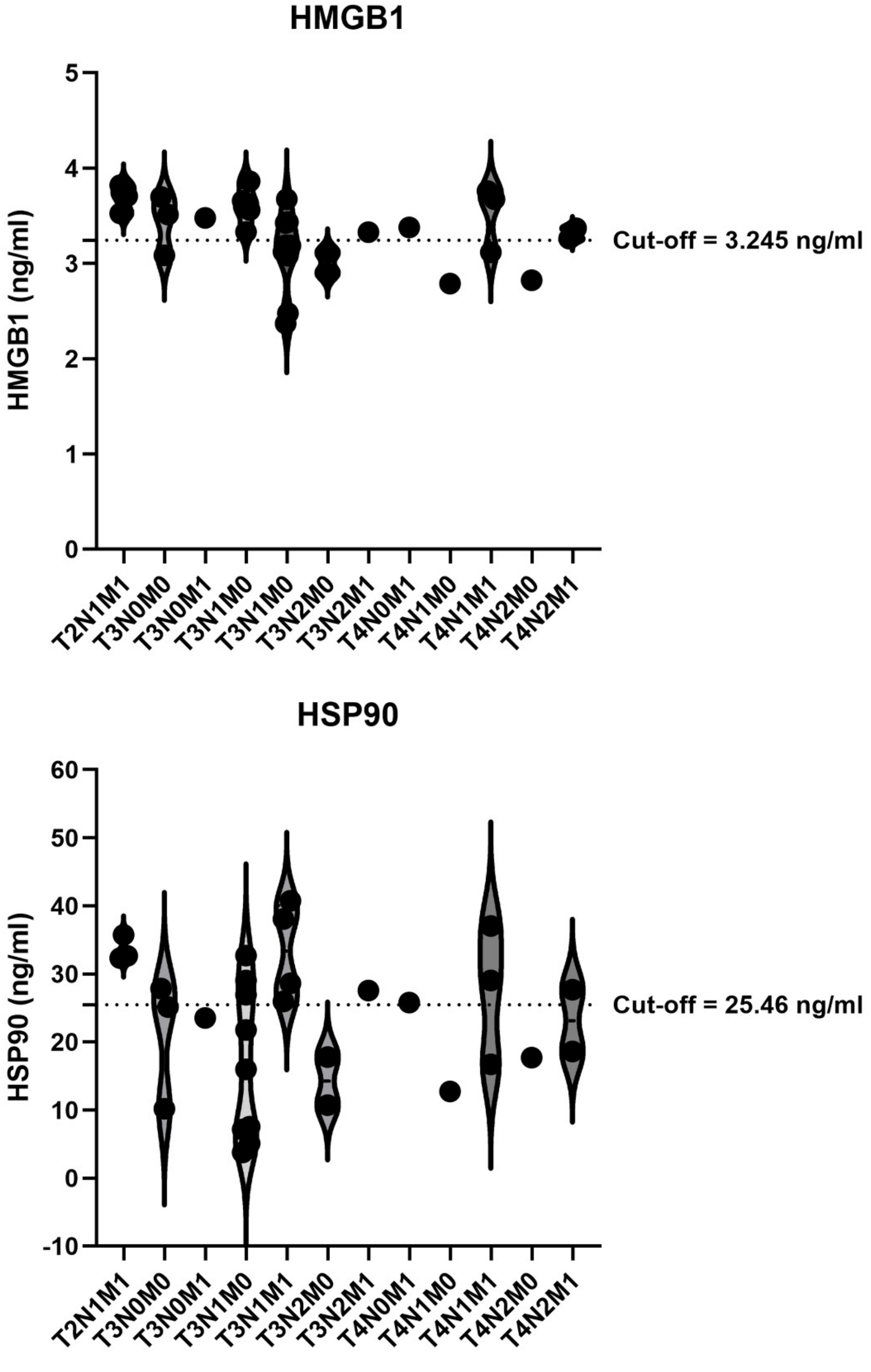

Figure S1. Mult-TNM analysis of cancer patients with HMGB1 and HSP90 levels.
